# Using clinical variables and drug prescription data to control for confounding in outcome comparisons between hospitals

**DOI:** 10.1186/s12913-014-0495-3

**Published:** 2014-10-23

**Authors:** Paola Colais, Mirko Di Martino, Danilo Fusco, Marina Davoli, Paul Aylin, Carlo Alberto Perucci

**Affiliations:** Department of Epidemiology, Regional Health Service, Lazio Region, Via Santa Costanza 53, 00198 Rome, Italy; School of Public Health, Imperial College London, South Kensington Campus, London, SW7 2AZ UK; National Outcome Program, Italian Agency for Health Services, Via Puglie 23, 00187 Rome, Italy

**Keywords:** Clinical variables, Drug prescription, Administrative data, Risk adjustment, Outcome research

## Abstract

**Background:**

Hospital discharge records are an essential source of information when comparing health outcomes among hospitals; however, they contain limited information on acute clinical conditions. Doubts remain as to whether the addition of clinical and drug consumption information would improve the prediction of health outcomes and reduce confounding in inter-hospital comparisons. The objective of the study is to compare the performance of two multivariate risk adjustment models, with and without clinical data and drug prescription information, in terms of their capability to a) predict short-term outcome rates and b) compare hospitals’ risk-adjusted outcome rates using two risk-adjustment procedures.

**Methods:**

Observational, retrospective study based on hospital data collected at the regional level.

Two cohorts of patients discharged in 2010 from hospitals located in the Lazio Region, Italy: acute myocardial infarction (AMI) and hip fracture (HF). Multivariate logistic regression models were implemented to predict 30-day mortality (AMI) or 48-hour surgery (HF), adjusting for demographic characteristics and comorbidities plus clinical data and drug prescription information. Risk-adjusted outcome rates were derived at the hospital level.

**Results:**

The addition of clinical data and drug prescription information improved the capability of the models to predict the study outcomes for the two conditions investigated. The discriminatory power of the AMI model increases when the clinical data and drug prescription information are included (c-statistic increases from 0.761 to 0.797); for the HF model the increase was more slight (c-statistic increases from 0.555 to 0.574). Some differences were observed between the hospital-adjusted proportion estimated using the two different models. However, the estimated hospital outcome rates were weakly affected by the introduction of clinical data and drug prescription information.

**Conclusions:**

The results show that the available clinical variables and drug prescription information were important complements to the hospital discharge data for characterising the acute severity of the patients. However, when these variables were used for adjustment purposes their contribution was negligible. This conclusion might not apply at other locations, in other time periods and for other health conditions if there is heterogeneity in the clinical conditions between hospitals.

**Electronic supplementary material:**

The online version of this article (doi:10.1186/s12913-014-0495-3) contains supplementary material, which is available to authorized users.

## Background

Over the last two decades, there has been increasing interest in the development of performance indicators in the attempt to promote accountability in health services [1-3]. These measures may concern different aspects of the system and reflect different objectives. “Process” measures, as surrogate outcome indicators, have been used to assess whether specific care processes recommended in clinical guidelines are administered, such as intervening within 48 hours of a hip fracture (HF). “Outcome” measures, such as the 30-day mortality rate after hospital admission for acute myocardial infarction (AMI), have been used to evaluate the effectiveness of health care processes.

In this respect, hospital discharge records have been an essential source of information for comparing health outcomes among hospitals [4]. They are widely available and represent a cost-effective source of information for monitoring health care quality in clinical practice over large populations and across a wide variety of conditions and procedures [5,6]. However, the amount of patient-level information collected in these archives is limited, generally consisting of age, gender, discharge diagnoses and main procedures. These data do not allow observers to characterise the acute clinical severity of the patient, but only to identify specific diseases as “comorbidities” (e.g., chronic pre-existing conditions that increase the a priori risk that the subject will incur adverse short-term health outcomes) [7,8]. For these reasons, the ability of administrative data to adjust for the severity of illness and to provide unbiased estimates of expected mortality rates at the hospital level has been criticised [9].

On the opposite extreme, clinical or laboratory data abstracted from medical records, when available, represent a good alternative because they may better account for the pre-hospital or pre-operative severity of illness, they may distinguish comorbidities (conditions already present at the time of admission or the procedure) from complications (conditions arising during hospitalisation or during the procedure) and they do not limit the number of reported diagnoses, so they avoid differential reporting of conditions according to the baseline severity of the patient [10,11]. The main drawbacks of the clinical archives are that their reliability may differ between hospitals and that it is difficult to obtain a large amount of clinical data at an affordable cost. Therefore, it is difficult to implement risk-adjustment methods based on these data in a systematic way [5,10].

Many authors and physicians advocate integrating both types of data to take full advantage of their relative strengths [12]. However, this strategy is often not feasible in clinical practice because of the difficulty obtaining timely and complete information on acute clinical severity. Thus, some investigators [13] have questioned whether it is possible to identify a limited number of laboratory or clinical data points that would be affordable and easy to collect from electronic medical archives and could be used to improve risk adjustment of inpatient mortality for different clinical conditions or procedures. In other words, as Johnston and colleagues asked, “Is there a low-cost way to improve the risk adjustment of administrative data?” [5].

Following these principles, medical professionals from different clinical areas and public health authorities began an audit activity in 2006 in the Lazio Region, Italy, with the aim of complementing the Hospital Information System (HIS) with a few selected clinical variables chosen to better characterise the acute severity of patients admitted for specific conditions. Ultimately, Coronary Artery Bypass Grafting (CABG), AMI and HF were selected, and a few clinical or laboratory data points were identified for each of them. The collection and transmission of this information became mandatory for all public and private hospitals in the Lazio region (where Rome is located) in 2008, and the new data became part of the new HIS.

Another information system with strong potential for comparative effectiveness research is the Regional Drug-Dispensing Registry (PHARM), which includes individual records for each drug prescription dispensed in public and private pharmacies. Some studies have evaluated the use of pharmacy dispensing data for predicting healthcare utilisation [14] or to identify patients with chronic conditions [15] but not specifically to control for confounding.

However, there is extensive scientific literature on the performance of “a priori” comorbidity scores based on outpatient pharmacy dispensing data, such as the Chronic Disease Scores [16-18]. Despite their popularity, these indices have limited utility in controlling for confounding because, like all summary scores, they assume a fixed relationship between comorbidities and the outcome, even though this relationship is likely to differ between populations. In fact, the risk-adjustment process should involve the construction of empirical illness severity and comorbidity measures specific to the study population. From this perspective, the scientific literature currently has significant gaps in terms of the evaluation of different types of empirical models for risk-adjustment procedures.

The integration of diagnosis-based models with medication-based predictive models is expected to result in greater predictive power and more exhaustive control of confounding, by modelling the complex relationships between diagnosed comorbidities and the presence of any pharmacological therapy, taking into account its benefit and harms. Extending these approaches to different diseases and conditions for developing and applying risk-adjustment models can provide new evidence to evaluate whether data on hospital performance are credible or methodologically flawed.

In the present study, we analysed two indicators, 30-day mortality after AMI admission and surgery within 48 hours after HF admission, with two objectives: 1) to compare a risk-adjustment model based on hospital discharge data only with a model including clinical variables and drug prescription information and 2) to investigate whether the two risk-adjustment procedures lead to different conclusions about hospital comparisons.

## Methods

### Data sources

The Lazio Region Hospital Information System (HIS) collects and manages data on all hospitalisations (registered in the Hospital Discharge Record, HDR) that have occurred since 1994 in the Lazio Region, an Italian region located in central Italy with ∼ 5 700 000 residents [19]. The HDR contains information on 172 hospitals (65 public hospital corporations, 95 private hospitals and 12 teaching hospitals) regarding patient characteristics (gender, place/date of birth, residence, etc.), hospital admission (date of admission, code of admitting hospital, admitting ward/specialty division, origin of patient, etc.), in-hospital transfers (dates and wards/divisions involved), discharge (date, discharging ward, type of discharge, etc.), clinical characteristics of the patient at discharge (main diagnosis +5 secondary diagnoses), and procedures performed during the hospital stay (main +5 secondary, with dates).

The New Information System collects data on clinical variables for all hospitalisations related to AMI and HF that have occurred in the Lazio Region since 2008. In particular, for all hospitalisations with a diagnosis code of AMI (ICD9-CM: 410.xx), the patient’s systolic blood pressure at the time of hospital admission is recorded. For all hospitalisations with a diagnosis code of HF (ICD9-CM: 820.xx), the pre-operative creatinine level (mg/dl), the value of the International Normalised Ratio (INR) and the time of hospital admission are registered. These additional clinical data were derived from a review process conducted by a panel of relevant clinical experts.

Drug utilisation data were available from the Regional Drug-Dispensing Registry (PHARM), which comprises individual records for each medical prescription dispensed in public and private pharmacies belonging to the regional health authorities and referring to residents. The registry is limited to drugs that are reimbursed by the health care system for outpatients. Drugs are identified by the national drug register code, which refers to the international Anatomical Therapeutic Chemical Classification System (ATC) and allows for the exact quantification of the dispensed drug. Individual patient data and drug-dispensing dates are reported for every prescription. Drugs dispensed were linked using the individual identification codes.

The study was conducted with the permission of the Department of Epidemiology of Lazio Regional Health Service, the regional referral centre for epidemiological research who has full access to anonymized hospitalization and drug prescription data therefore ethics approval was not required.

### Study population

#### AMI cohort

The AMI cohort consists of all patients who were hospitalised in the Lazio Region between January 2010 and November 2010 with a primary diagnosis of AMI (ICD9-CM = 410.xx) or a secondary diagnosis of AMI and a complication of myocardial infarction as the primary diagnosis (ICD9-CM diagnosis codes: 423.0, 427.0, 427.1, 427.2, 427.3, 427.4, 427.6, 427.8, 427.9, 429.5, 429.6, 518.4, 780.2, 799.1, 998.2). If the same subject was re-hospitalised for AMI within 28 days of the first hospital admission, only the first admission was included, under the assumption that the following admissions were part of the same AMI episode. Furthermore, only subjects aged 18–100 years who resided in the study area were included, and only records with complete administrative and clinical data were retained to guarantee complete comparability of the risk-adjustment models.

For all subjects, information was available on the following parameters: age, gender, systolic blood pressure and a list of co-morbidities chosen a priori and defined on the basis of the primary and secondary diagnoses for all hospital admissions that occurred in the previous two-year period [20,21]. On the contrary, secondary diagnoses from the index hospitalisations were considered only when they did not refer to the diagnoses at discharge to distinguish between pre-existing conditions and complications that arose after admission. (The complete list of co-morbidities and details on the ICD9-CM codes are available on request).

For all patients, information on drug prescriptions was available from the PHARM registry. We collected information on anticoagulants (ATC codes: B01AA and B01AB), antiplatelet agents (ATC: B01AC), cardiac therapy drugs (C01), antihypertensive drugs (C02), diuretics (C03), beta-blocking agents (C07), calcium channel blockers (C08), ACE inhibitors (C09A and C09B), angiotensin II antagonists (C09C and C09D), statins (HMG CoA reductase inhibitors, C10AA), other lipid-modifying agents (C10 excluding C10AA), and anti-diabetic drugs (A10). Treatment was defined as at least 1 prescription in the 3 months preceding the AMI admission.

#### HF cohort

The HF cohort includes all patients hospitalised in the Lazio Region between January 2010 and November 2010 with a primary or secondary diagnosis of HF (ICD9-CM = 820.xx). The following exclusion criteria were applied: 1) subjects with HF in the previous two years; 2) subjects younger than 65 years or older than 100 years; 3) patients residing outside the Lazio Region; 4) hospitalisations with a diagnosis of cancer (ICD9-CM: 140–208) on the index admission or in the previous two years; 5) patients with multiple trauma (Diagnosis Related Groups - DRG codes: 484–487); 6) hospitalisations in which the patient was admitted directly to an intensive care unit; and 7) hospitalisations in which surgery was not performed and the patient died within the first 48 hours. Furthermore, only records with complete administrative and clinical data were retained to guarantee complete comparability of the risk-adjustment models.

For all the subjects, information was available on age, gender, clinical variables, and an extensive list of co-morbidities [20,22] chosen a priori and defined on the same basis as reported for AMI cohorts.

For all patients, information on drug use was available from the PHARM registry. We collected information on anticoagulants (ATC codes: B01AA, B01AB) and antiplatelet agents (ATC: B01AC), according to the specific hypothesis that the presence of these drug treatments could delay anaesthesia and, consequently, the surgery. Treatment was defined as at least 1 prescriptions in the 3 months preceding the HF admission.

### Study outcomes

We defined two different outcomes:

1. Mortality within 30 days after AMI admission. Deaths during the study period were identified using both the HIS (discharge disposition: death) and the Mortality Information System (MIS).
2. The proportion of interventions performed within 48 hours (0–1 day) of HF admission. The date and time of hospital arrival corresponded to the date and time of the index admission.

### Statistical analysis

#### Risk-adjustment models

Multivariate logistic regression models were built for the study outcomes beginning with the hospital discharge data only to identify the relevant risk factors among the variables collected from the HIS. In particular, age and gender were a priori considered risk factors for all the study outcomes and were thus included in the risk-adjustment models regardless of their actual associations with the outcomes. For the co-morbidities, a bootstrap stepwise procedure that assigned an importance rank for the predictors in the logistic regression was implemented to identify the set of conditions that significantly predicted the risk of the outcome while optimising the trade-off between the goodness-of-fit of the final model and parsimony. Using this approach, the logistic regression with all predictors was run 1000 times on random samples drawn with replacement from the original data set. Only the risk factors identified as significant (p ≤ 0.05) at least 30 times in at least 30% of the procedures were included in the predictive model. These steps were used to define the “hospital discharge data” model.

The “hospital discharge data + clinical variables + drug prescriptions” model was built in the same way using a bootstrap stepwise procedure and adding the available clinical variables and the drug prescription variables to the set of variables collected from the HIS.

The two risk-adjustment models were compared for each of the two study outcomes by analysing their discriminatory power using the “c” statistic, which has been demonstrated to be equivalent to the area under the receiver-operating characteristics (ROC) curve [23].

#### Hospital comparison

To compare outcomes attributed to different hospitals, we applied the direct standardisation method by creating a multivariate logistic regression with no intercept and centred covariates.

This model estimates the log odds of 30-day mortality and an intervention within 48 hours by hospital. Adjusted proportions were obtained for each hospital by back-transforming parameter estimates using the following formula [24]:

$$ \mathrm{A}\mathrm{d}\mathrm{j}\ \mathrm{proportion} = \kern0.5em \left[ \exp \left(\mathrm{estimate}\right)\ /\ \left(1 + \exp \left(\mathrm{estimate}\right)\right)\right]*\mathrm{k} $$

Where k is a correction coefficient introduced to account for the non-linear nature of the logistic model.

K is calculated as follows:

$$ \mathrm{K} = \mathrm{actual}\ \mathrm{number}\ \mathrm{of}\ \mathrm{events}/{\displaystyle \sum_{j=1}^m{p}_j*{n}_j} $$

Where p_j_ is the adjusted proportion, n_j_ is the group size, and *m* is the number of groups.

The adjusted proportions for each hospital were plotted on a funnel plot in which the observed indicator was plotted against a measure of its precision so that the control limits form a ‘funnel’ around the target outcomes (overall 30-day mortality after AMI admission and the proportion of interventions performed within 48 hours from HF admission).

A sensitivity analysis using multilevel logistic regression with a random intercept, for both AMI and HF cohort, was performed in order to compare the findings deriving from the two different approaches.

All statistical analyses were conducted using SAS, version 9.2 [25].

## Results

### Cohort characteristics

The distributions of patient characteristics according to hospital discharge data, clinical variables and drug prescription data are reported for the two study cohorts in Table 1.

**Table 1:** **Distribution of demographic characteristics, chronic conditions, clinical variables and drug prescriptions**

**Risk factor**	**AMI**		**HF**	
	**(No. 7613)**		**(No. 6348)**	
	**No.**	**%**	**No.**	**%**
*Demographic characteristics*				
Age (mean, SD)	70.1*	13.5*	83.0*	7.1*
Gender (female)	2690	35.3	4925	77.58
*Previous conditions (from HIS)*				
Cancer	437	5.74		
Diabetes	829	10.89	406	6.4
Nutritional deficiencies			18	0.28
Lipid metabolism disorders	344	4.52		
Obesity	74	0.97	23	0.36
Blood disorders	338	4.44	322	5.07
Dementia including Alzheimer’s disease			192	3.02
Parkinson’s disease			56	0.88
Hemiplegia and other paralytic syndromes			22	0.35
Rheumatic heart disease	67	0.88	30	0.47
Hypertension	1382	18.15	877	13.82
Previous myocardial infarction	1137	14.93	163	2.57
Other forms of ischemic heart diseases	998	13.11	560	8.82
Acute endocarditis and myocarditis	2	0.03		
Cardiomyopathy	95	1.25	50	0.79
Conduction disorders and arrhythmias	592	7.78	479	7.55
Heart failure	554	7.28	356	5.61
Ill-defined descriptions or complications of heart disease	137	1.8	117	1.84
Other heart conditions	103	1.35	82	1.29
Cerebrovascular disease	512	6.73	561	8.84
Vascular disease	304	3.99	154	2.43
Chronic obstructive pulmonary disease (COPD)	423	5.56	366	5.77
Chronic diseases (liver, pancreas, intestine)	85	1.12	71	1.12
Chronic renal disease	419	5.5	232	3.65
Rheumatoid arthritis and other inflammatory polyarthropathies			25	0.39
Osteoporosis and other disorders of bone and cartilage			57	0.9
Previous coronary artery bypass graft	310	4.07		
Previous coronary angioplasty	854	11.22		
Cerebral revascularisation procedures	42	0.55		
Other cardiac operations	47	0.62		
Other vascular operations	182	2.39		
Clinical data (from the New Information System)				
Systolic blood pressure				
≤ 100 mmHg	765	10.05		
> 100 mmHg	6592	86.59		
Missing	256	3.36		
International Normalised Ratio (INR)				
0.9-1.2			4,834	76.15
Out of range			855	13.47
Missing			659	10.38
Drug prescriptions^a^				
Anticoagulants	493	6.48		
Antiplatelet agents	2447	32.14		
Cardiac therapy drugs	1686	22.15		
Antihypertensive drugs	359	4.72		
Diuretics	1577	20.71		
Beta-blocking agents	1554	20.41		
Calcium channel blockers	1570	20.62		
ACE inhibitors	1977	25.97		
Angiotensin II antagonists	1823	23.95		
Statins	1885	24.76		
Other lipid-modifying agents	557	7.32		
Anti-diabetic drugs	1614	21.2		
Antiplatelet (3 months)			851	13.41
Anticoagulants (3 months)			238	3.75

*Mean and standard deviation.

^a^Treatment was defined as at least 1 prescription in the 3 months preceding the AMI/HF admission.

The AMI cohort consisted of 7613 episodes treated in 62 hospitals: 42 public hospital corporations, 15 private hospitals and 5 teaching hospitals. There are no substantial differences in patient characteristics between type of hospitals as shown in (see Additional file 1). A total of 29 (0.38%) records with not complete administrative and clinical data were excluded. The crude 30-day mortality rate was 10.8%. The mean age was 70 years, with a small proportion of women (35%). Among the selected chronic conditions, based on the hospitalisations in the previous two years, the most frequent conditions were hypertension (18%), previous myocardial infarction (15%), diabetes and other forms of ischemic heart diseases (13%). The only clinical variable was the systolic blood pressure (SBP) collected at the time of hospital admission, and 10% of the AMI episodes were characterised by SBP values below 100 mmHg. The drug prescription variables included diuretics (21%), ACE inhibitors (26%) and angiotensin II antagonists (24%) prescribed in the three months preceding the hospital admission.

The HF study population consisted of 6348 hospitalisations treated in 83 hospitals: 42 public hospital corporations, 34 private hospitals and 7 teaching hospitals. There are no differences in patient characteristics between type of hospitals as shown in (see Additional file 2). A total of 10 (0.16%) records with not complete administrative and clinical data were excluded. The crude proportion of interventions performed within 48 hours was 19.8%. The characteristics of the HF cohort were different from those of the AMI cohort: the population was older (65–100 years of age) and included more women (78%). The list of comorbidities was more extensive: the most frequent were hypertension (14%), cerebrovascular disease (9%), other forms of ischemic heart diseases (9%), conduction disorders and arrhythmias (8%) and diabetes (6%). 13% of the patients reported an out-of-range INR value.

### Comparison of risk-adjustment models

The risk-adjustment models for AMI and HF are displayed in Tables 2 and 3, respectively. Each table reports the variables retained in the final risk-adjustment models, with the corresponding number of admissions, crude ORs and adjusted ORs from the “hospital discharge data” model and the “hospital discharge data + clinical variables + drug prescriptions” model. Finally, the results from the c-statistics are reported.

**Table 2:** **Predictive models for 30-day mortality after AMI admission (No. 7613)**

**Risk factor**	**n (admissions)**	**Crude OR**	**Hospital discharge data**		**Hospital discharge data + clinical variables + drug prescriptions**	
			**Adjusted OR**	**P**	**Adjusted OR**	**P**
Age	-	1.08	1.08	0.000	1.08	0.000
Gender (females vs males)	2690	1.66	0.97	0.738	0.96	0.589
Cancer	437	1.87	1.44	0.008	1.42	0.013
Diabetes	829	1.54	1.34	0.013		
Disorders of lipid metabolism	344	0.42	0.42	0.001	0.46	0.004
Blood disorders (index admission)	393	1.39			0.69	0.027
Blood disorders	338	2.34			1.28	0.127
Previous AMI	1137	0.85	0.76	0.033	0.71	0.010
Heart failure	554	2.55	1.66	0.000	1.51	0.003
Other forms of ischemic heart disease (index admission)	175	0.88	0.48	0.009	0.47	0.008
Other forms of ischemic heart disease	103	1.41	1.33	0.384	1.26	0.478
Chronic renal disease	419	2.68	1.65	0.000	1.48	0.007
Other chronic disease (liver, pancreas, intestine)	85	2.08			1.92	0.035
Previous CABG	310	0.50	0.50	0.007	0.52	0.012
Previous PCI	854	0.47	0.61	0.004	0.62	0.006
Blood pressure > 100	6592	1.00			1.00	-
Blood pressure < =100	765	4.07			4.60	0.000
Blood pressure missing	256	1.27			1.33	0.195
Diuretics (3 months)	1577	2.54			1.69	0.000
ACE inhibitors (3 months)	1977	1.12			0.77	0.005
Angiotensin II antagonists (3 months)	1823	1.01			0.82	0.039
*Area under ROC curve*			*0.761*		*0.797*

**Table 3:** **Predictive models for surgery performed within 48 hours of HF admission (No. 6348)**

**Risk factor**	**n (admissions)**	**Crude OR**	**Hospital discharge data**		**Hospital discharge data + clinical variables + drug prescriptions**	
			**Adjusted OR**	**P**	**Adjusted OR**	**P**
Age	-	1.00	1.00	0.420	1.00	0.456
Gender (females vs males)	4925	1.42	1.40	0.000	1.37	0.000
Diabetes	406	0.66	0.72	0.036	0.68	0.011
Obesity (index admission)	41	2.36	2.21	0.016	2.33	0.011
Obesity	23	1.13	1.22	0.705	1.04	0.935
Hypertension	877	0.76	0.80	0.028		
Osteoporosis	57	2.05	2.13	0.008	1.97	0.017
INR 0.9-1.2	4834	1.00				-
INR out of range	855	0.57			0.56	0.000
INR missing	659	0.83			0.78	0.023
Antiplatelet (3 months)	851	0.78			0.79	0.020
*Area under ROC curve*			*0.555*	*0.574*		

#### AMI cohort

Age was a strong predictor of 30-day mortality, with an adjusted excess risk of dying of 8% for each one-year increase in age, in both the “hospital discharge data” and the “hospital discharge data + clinical variables + drug prescriptions” models. On the contrary, no gender differences emerged, even after adjusting for age, comorbidities, clinical variables and drug prescriptions. A long list of comorbidities were included in the final risk-adjustment model; other chronic diseases (liver, pancreas, intestine), heart failure, chronic renal disease, cancer and other forms of ischemic heart disease had the strongest associations with mortality. Blood pressure as measured at the time of hospital admission is strongly associated with 30-day mortality (risk-adjusted OR = 4.60, p-value < 0.001). Interestingly, the OR from the risk-adjusted model is almost identical to the OR from the crude model, meaning that the inclusion of past conditions does not alter the predictive power of the SBP. In other words, it seems that this parameter captures a different dimension of the severity of the patient’s condition (e.g., acute severity) from that measured by comorbidities (chronic severity). With regard to the use of drugs in the 3 months preceding the AMI admission, the bootstrap stepwise procedure identified three variables that were significantly associated with the outcome: the use of diuretics, the use of ACE inhibitors and the use of angiotensin II antagonists. The effects of these drugs on 30-day mortality are discordant: while diuretics act as a risk factor (adjusted odds ratio: 1.69), ACE inhibitors and angiotensin II antagonists act as protective factors (adjusted odds ratios: 0.77 and 0.82, respectively).

As a consequence, the discriminatory power of the model increases when the clinical data and drug prescription information are included (c-statistic increases from 0.761 to 0.797).

#### HF cohort

Age was not associated with the outcome in either the crude model or the adjusted models; however, gender differences emerged, even after adjusting for age, comorbidities, clinical variables and drug prescriptions. Only diabetes, obesity and osteoporosis were included in the final model from the extensive list of conditions defined a priori. The proportion of patients who received surgery within the expected 48 hours was half as high for patients with out-of-range INR compared with patients with in-range values. With respect to the variables extracted from the PHARM registry, only the use of antiplatelet agents was selected in the bootstrap stepwise procedures. Previous users of platelet aggregation inhibitors were less likely to receive surgery within 48 hours of hospital admission.

The two risk-adjustment models (“hospital discharge data” VS “hospital discharge data + clinical variables + drug prescriptions”) performed equally poorly: the discriminatory power of the two models is only slightly higher than what would have been expected by chance (c = 0.5), with a slight increase in the c-statistic after the clinical variables and drug prescriptions were taken into account (c-statistic increased from 0.555 to 0.574).

### Hospital comparison

**Figure 1:**
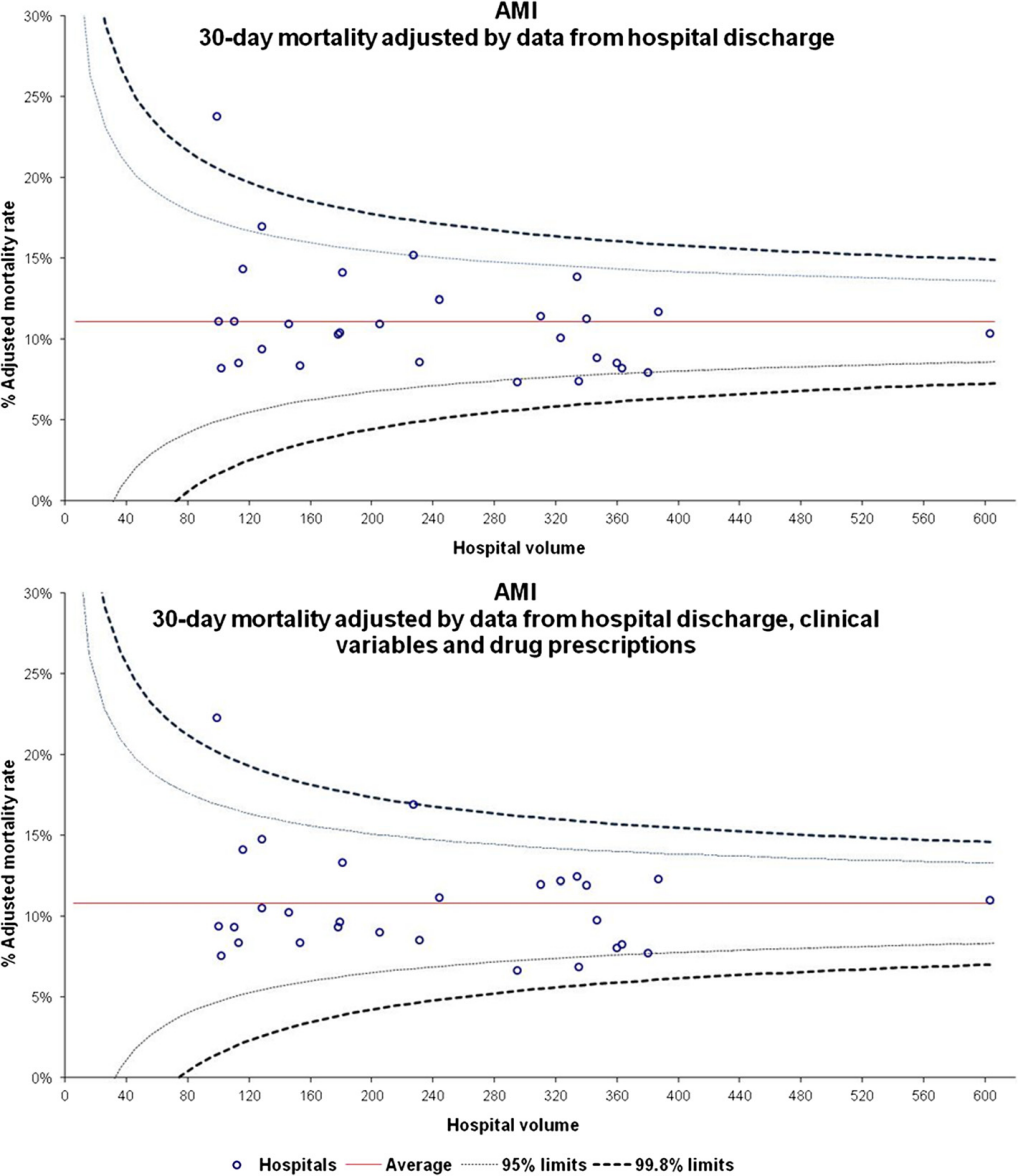
**Adjusted 30-day mortality after AMI admission, by hospital (No. 7613).**

In Figure 1, the hospital-adjusted proportion of 30-day mortality after AMI admission estimated using the “hospital discharge data” model and the hospital-adjusted proportion estimated using the “hospital discharge data + clinical variables + drug prescriptions” model are plotted in a funnel plot.

**Figure 2:**
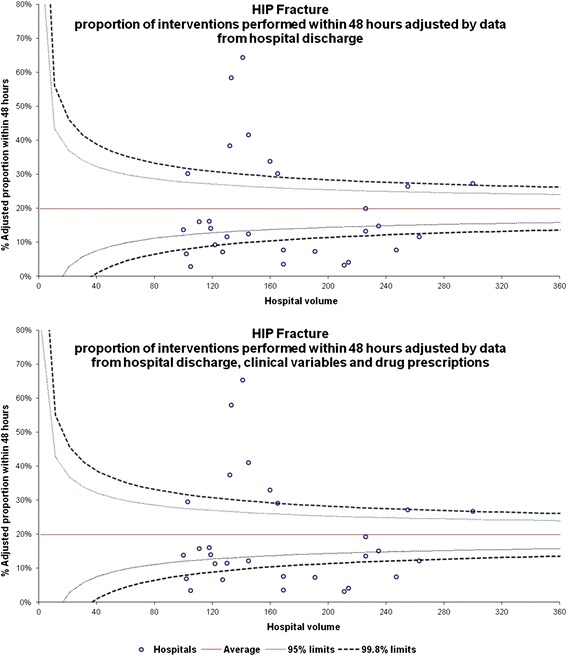
**Adjusted proportion of interventions performed within 48 hours of HF admission, by hospital (No. 6348).**

In Figure 2, the hospital-adjusted proportion of patients who received an intervention within 48 hours after HF admission estimated using the “hospital discharge data” model and the hospital-adjusted proportion estimated using the “hospital discharge data + clinical variables + drug prescriptions” model are plotted in a funnel plot.

#### AMI cohort

The observed mortality rates ranged from 5.4% to 22.2%. The variability of the adjusted mortality rates from the two models was different in magnitude, ranging from 6.7% to 22.3% in the “hospital discharge data” model and from 7.3% to 23.8% in the “hospital discharge data + clinical variables + drug prescriptions” model.

Some differences were observed between the hospital-adjusted proportion estimated using the “hospital discharge data” model and the hospital-adjusted proportion estimated using the “hospital discharge data + clinical variables + drug prescriptions” model (Figure 1). However, the funnel plots clearly reveal that the bulk of hospitals lie within the 95% limits, and only one hospital lies outside the 99.8% limits.

#### HF cohort

The observed proportion of patients who received an intervention within 48 hours ranged from 2.9% to 63.8%. The variability of the adjusted proportion from the two models was different in magnitude, ranging from 2.9% to 64.4% in the “hospital discharge data” model and from 3.2% to 65.3% in the “hospital discharge data + clinical variables + drug prescriptions” model. As expected in the risk-adjustment models, there was very close agreement between the observed proportions of surgery within 48 hours and the expected proportions derived from the two risk-adjustment models; both models were not able to characterise the pre-operative probability of an intervention at the patient level. Thus, the funnel plots clearly revealed the same pattern when the hospital-adjusted proportion estimated using the “hospital discharge data” model and the hospital-adjusted proportion estimated using the “hospital discharge data + clinical variables + drug prescriptions” model were plotted (Figure 2).

With regard to the sensitivity analysis, multilevel and fixed-effects models lead to similar results: both random and fixed effects analyses showed that adding clinical variables to the adjusting models did not modify the hospital ranking.

## Discussion

The present study was designed to quantify the additional contribution of clinical variables and drug prescription information in predicting short-term outcome rates and profiling hospitals compared to the use of hospital discharge data alone. We found that the risk adjustment improved considerably for the AMI cohort after the three sources of data were integrated, whereas the two risk-adjustment models performed equally poorly for the HF cohort. However, hospital profiling was not affected by the use of clinical variables and drug prescriptions for the HF cohort, whereas for the AMI cohort, some differences in hospital profiles were observed even if the “low performing” and the “best performing” institutions were the same regardless of the risk adjustment model applied.

The optimal approach to producing hospital outcome reports relies on collecting valid information to provide an accurate risk adjustment. This approach requires medical chart abstraction, which is expensive and therefore has not been widely implemented by public reporting agencies. Using administrative data for public outcomes reporting offers several advantages, including minimal data collection costs and the ability to produce reports for a large number of procedures and conditions [26]. However, these data do not capture important clinical information about the acute severity of the patient and do not distinguish between the conditions that were present at admission and the complications that occurred during hospitalisation [7,27,28]. Many authors have advocated for identifying a limited number of affordable and easily accessible laboratory or clinical data points from electronic medical archives that would improve risk-adjustment models of inpatient mortality for different clinical conditions or procedures [12]. On this basis, in the present study, we used clinical information from the upgraded version of the HDR, including a few selected clinical variables chosen to better characterise the acute severity of patients admitted for AMI and HF. The Lazio HIS information is widely available and high in quality, and it represents a highly cost-effective solution for monitoring health care quality in clinical practice over large populations and across a wide variety of conditions and procedures.

The conditions to integrate from the discharge abstracts and the clinical variables to add were derived from an extensive audit activity that was conducted beginning in 2006 in the Lazio Region, Italy by medical professionals from different clinical areas and public health authorities. They opted for AMI and HF, conditions that pose significant public health problems, and identified a few clinical parameters (blood pressure and INR) that could be detected at affordable costs and are considered valid and reliable markers of acute severity.

The present study has some important strengths. It is the first study conducted in Italy with the specified aim of comparing the performance of risk-adjustment models with and without clinical variables and drugs prescription information in the context of hospital profiling and comparative outcomes research in general. The high number of patients investigated, the accuracy in the selection of the cohorts and the study outcomes, the consolidated statistical strategy, and the replication of similar findings for different clinical conditions are important elements of internal and external validity.

A limitation of this study is the generalizability of our results due to selection of only hospitalizations from Lazio region, however the large number of residents and hospitals in this region minimize the variability of case-mix between the admission in Lazio hospitals and in other Italian hospitals. Other limitations should also be acknowledged, especially the marked variability in the coding accuracy of current health care information systems. This issue is critical for ensuring accurate risk adjustment and thus reliable comparative quality ratings [29]. However, data derived from health information systems are currently utilised to compare inpatient care outcomes in Italy [30,31] and have proved to be an accurate source for healthcare research and a reliable data source for adjusting for risk factors [8,32].

Moreover, as in the case of HF, risk-adjustment models may not be able to predict the study outcome (surgical treatment), even when they include valid clinical information on the severity of disease and drug prescriptions. This result simply means that the determinants of the outcome should be sought among the characteristics of the hospital and health care, which is the eventual purpose of hospital comparisons.

More generally, in outcome comparisons between hospitals, where each hospital represents a level of exposure, potential clinical confounders cannot produce important changes in the adjusted measures of association if these factors are not heterogeneously distributed between hospitals, even when they are strongly associated with the outcome under study.

## Conclusions

In conclusion, the present study represents the first effort in Italy to compare the performance of risk-adjustment procedures incorporating clinical variables and drug prescriptions in predicting short-term outcomes and in profiling hospitals on the basis of the predicted outcomes. We found that the available clinical variables and drug prescription information were important complements to the hospital discharge data for characterising the acute severity of the patients for one of the two conditions we analysed. However, when the output of the predictive models was used to compare the hospitals on the basis of their risk-adjusted outcomes, the contribution of the clinical variables and drug prescriptions was always negligible. We hope that this approach will be replicated in other studies, for other clinical conditions, and for different clinical parameters and alternative analytical procedures to better interpret the present results and to better understand the trade-off between the costs and the advantages of including relevant clinical variables in systematic health information systems.

### Data used for the study

The data used for the study are not openly available. The Department of Epidemiology has been authorised by the Regional Health Authority to use the data.

## Additional files

Additional file 1:
**Distribution of demographic characteristics, chronic conditions, clinical variables and drug consumption by type of hospitals - AMI cohort.**
Additional file 2:
**Distribution of demographic characteristics, chronic conditions, clinical variables and drug consumption by type of hospitals - HF cohort.**
